# A mixed methods implementation evaluation of a brief movement-based health promotion workshop for health professionals in the Ecuadorian highlands

**DOI:** 10.3389/fpubh.2026.1834768

**Published:** 2026-05-11

**Authors:** Dawn M. Magnusson, Pablo Boada, Lilly S. Webster, Carlos Quezada

**Affiliations:** 1 Physical Therapy Program, Department of Physical Medicine and Rehabilitation, University of Colorado School of Medicine, Aurora, CO, United States; 2 Fundación Hombro a Hombro, Quito, Ecuador

**Keywords:** community health worker, Ecuador, health equity, health promotion, health workforce training, indigenous health, Latin America, physical activity counseling

## Abstract

**Background:**

Although Ecuador’s Ministry of Public Health promotes regular physical activity, program design and implementation remains limited, especially in rural Indigenous communities. The purpose of this study was to examine Movimiento como Medicina (MCM) as a contextually grounded implementation strategy to enhance movement and physical activity counseling among health professionals serving Indigenous communities.

**Methods:**

An exploratory mixed methods design was used to assess core implementation outcomes: acceptability, appropriateness, and feasibility. Pre-post changes in provider confidence served as a proximal indicator of adoption. Differences in confidence were analyzed using a two-tailed Wilcoxon Signed-Rank Test for paired, non-parametric data. Qualitative data, collected via text responses to open-ended survey questions and analyzed via deductive thematic content analysis, helped further elucidate participants’ assessment of the program.

**Results:**

Thirty-six health professionals completed the one- or two-day MCM workshop. The mean acceptability score was 4.8 (Standard Deviation [SD] = 0.69). Ninety percent or more of participants strongly agreed that the training objectives were clearly stated, the facilitators were knowledgeable about the topics and created an engaging learning environment, and the pace of the training was appropriate. The mean appropriateness score was = 4.8 (SD = 0.68). Ninety-three percent of participants strongly agreed that the content was relevant to their work and that the program increased their understanding of movement as medicine. Seventy-seven percent of participants strongly agreed that the training included culturally appropriate examples. The most useful aspects of the training included talking through and applying content to learning cases and practicing the various movement-based strategies. The mean feasibility score was 4.65 (SD = 0.61). 92% of participants were likely or very likely to integrate movement strategies into their practice as a result of the workshop. Participants’ confidence in identifying the four components of movement, discussing the benefits of movement, and exploring community beliefs regarding movement increased significantly from pre- to post-training.

**Conclusion:**

MCM’s high acceptability, appropriateness, and feasibility, combined with significant improvements in participant confidence, positions the program as a contextually aligned and scalable health promotion strategy.

## Introduction

Despite Ecuador’s national commitment to health promotion and physical activity (1), translating evidence-based recommendations into routine practice remains challenging, especially within Indigenous-serving health systems. In Ecuador, 1.3 million Indigenous Peoples from 14 nationalities reside primarily in the Highlands (68%) and the Amazon (24%) (2). Despite strong cultural traditions and resilience, Indigenous communities face profound health inequities (3). Life expectancy is often 20 years lower than that of non-Indigenous populations, while rates of infant and maternal mortality, chronic health conditions, and disability remain disproportionately higher (4, 5). The unequal “epidemiological accumulation” of both acute and chronic disease burden is deeply rooted in colonial histories of dispossession and systemic marginalization and perpetuated through enduring social, economic, cultural, and political barriers (3, 4, 6, 7). Although these inequities are well-documented, limited attention has been paid to how evidence-based health promotion interventions can be integrated into local health systems.

Implementation science provides a useful framework for addressing this gap by distinguishing between evidence-based interventions (EBIs) and the implementation strategies used to support their uptake. Evidence-based clinical interventions are defined as a specific clinical/therapeutic practice, delivery arrangement, or health promotion activity being implemented to improve health outcomes, while implementation strategies are defined as a bundle of methods or techniques designed to enhance adoption of EBIs (8, 9). Evaluating implementation strategies is essential because EBIs – such as physical activity assessment, counseling, and prescription – often fail to achieve impact; not due to lack of efficacy, but due to their limited acceptability, appropriateness, or feasibility in real-world settings.

This distinction is especially salient in Indigenous settings where the successful integration of EBIs depends heavily on their alignment with local health systems, beliefs, and practices, and their delivery by local healthcare professionals. Community health workers (CHWs) play a critical role in bridging known implementation gaps (10). In Ecuador, CHWs play an essential role in identifying local priorities, co-developing health promotion solutions, and facilitating culturally-responsive care. Exploratory conversations with leaders in Cotopaxi and Pichincha Provinces highlighted the need to strengthen CHW and primary care provider capacity to implement evidence-based health promotion activities, paying particular attention to the prevention of heart disease, stroke, hypertension, diabetes, arthritis, and back pain. Such chronic conditions now surpass acute, infectious diseases as the leading causes of mortality and morbidity worldwide (11).

Physical activity, defined broadly as any movement that requires energy expenditure, reduces the risk and progression of non-communicable diseases while promoting physical and mental well-being (12). WHO recommends at least 150 min of moderate-intensity activity or 75 min of vigorous-intensity activity per week (12), yet 31% of adults do not meet these guidelines, with higher rates of inactivity among older adults (13). In Ecuador, 20%–30% of adults are inactive (13), and physical inactivity’s contribution to disability-adjusted life years has risen over the last three decades (14). The United States Preventive Services Taskforce recommends behavioral counseling within primary care settings to help increase physical activity (15, 16).

Movimiento como Medicina (MCM) is a contextually-grounded implementation strategy designed to strengthen providers’ capacity to deliver evidence-based physical activity recommendations (i.e., assessment, counseling, and prescription). There is limited published research describing how such programs are designed, implemented, and received by CHWs and primary care providers in Ecuador. Consistent with recommendations for reporting and evaluating implementation strategies (9), the purpose of this study was to examine the preliminary effectiveness of MCM as a contextually grounded implementation strategy, paying particular attention to its acceptability, appropriateness, feasibility, and proximal adoption.

## Methods

### Study design

Prior to large scale dissemination of MCM and subsequent hybrid effectiveness trials, we leveraged an exploratory, convergent mixed methods design (17) in this early-phase, community-based research study to assess core implementation outcomes: acceptability, appropriateness, feasibility, and proximal adoption. Quantitative and qualitative findings were collected and analyzed simultaneously and then integrated to compare findings and provide a more holistic assessment of the program. Such information is critical prior to larger scale implementation and effectiveness trials. The University of Colorado Multiple Institutional Review Board (26–3,032) has approved this study. All methods were carried out in accordance with relevant human subjects guidelines and regulations, and informed consent was obtained from participants prior to data collection.

### Setting

The study took place December 1–5, 2025 in Pichincha and Cotopaxi Provinces. These central Andean highlands are characterized by dramatic elevation changes, fertile volcanic soils, and a chain of inter-mountain valleys that have long-supported Indigenous communities with vibrant cultural traditions and place-based agricultural practices. Cayambe sits in the northeast corner of Pichincha Province. Indigenous Peoples comprise 35% of Cayambe’s population and its inter-mountain valley is known for its floriculture and global rose exports. Latacunga, the capital of Cotopaxi Province, lies in a broad valley south of Quito. The city itself is urban, with a mestizo majority, while the surrounding cantons (i.e., villages) are primarily Indigenous. The landscape is similar to Cayambe, displaying a patchwork of highland plains, river valleys, and pastoral lands. Smaller farms support traditional agriculture and dairy production that supply national markets, while larger vegetable farms support national markets and global supply chains. Despite high employment rates, low wages, chemical exposures, long workdays, and repetitive movements contribute to a number of chronic conditions that are exacerbated by limited access to preventive and comprehensive healthcare services.

### Participants and recruitment

MCM was initially designed for CHWs; however, conversations with leaders from the Ministerio de Salud Publica en Pichincha led to the adoption of the program for local healthcare providers. Thus, two participant groups (i.e., action targets) were enrolled in the study

- Group 1 consisted of general practice physicians, family and community physicians, nurses, physical therapists, occupational therapists, speech therapists, and CHWs from Pichincha Province who are employed within the public healthcare system. This group participated in a one-day, six-hour workshop. Given the limited availability of these individuals for the training, our team prioritized the learning objectives and activities that most closely aligned with providers’ needs and interests.
- Group 2 included CHWs and physical therapists from Cotopaxi Province who were employed within the Gobierno Provincial de Cotopaxi and Latacunga Patronato. This group participated in a two-day, 12-h workshop.

Participants were recruited through a combination of direct outreach and community-based communication strategies, led by the Hombro a Hombro (HH) Foundation. Our team partnered with the Ministerio de Salud Publica en Pichincha and the Gobierno Provincial de Cotopaxi to disseminate information about the training via direct messaging (WhatsApp). Details of the training opportunity were also shared during regularly-scheduled meetings and within established provider networks to help raise awareness. Interested individuals contacted their local coordinator (Pichincha Ministerio de Salud Publica or Cotopaxi Solidario) via WhatsApp to confirm their participation. Enrollment was open to all eligible healthcare providers and CHWs serving “Kichwas de la sierra” in the Pichincha and Cotopaxi Highlands. Prior participation in movement-based training programs did not preclude anyone from enrolling in MCM.

### Program components

Lee and colleagues’ scoping review of community health worker training programs (10) and the Template for Intervention Description and Replication (TIDieR) Checklist informed our selection of the following reporting elements.

#### Organizational structure

MCM was intentionally embedded within existing organizations and structures, recognizing the critical importance of local systems in shaping healthcare access and quality, professional development, and program sustainability. The workshops resulted from collaborative conversations among HH, leaders within the Gobierno Provincial de Cotopaxi and Cotopaxi Solidario, leaders within the Ministerio de Salud Publica in Pichincha province, and faculty from the University of Colorado Physical Therapy Program (CU-PT, United States of America). HH has collaborated with rural Indigenous communities throughout Ecuador for over a decade. Thanks to the participation of HH in an interprovincial meeting where they discussed MCM, interest in the program grew among officials within Pichincha Ministerio de Salud Publica.

#### Program resources and funding

MCM relied on a combination of in-kind contributions and local partnerships to support implementation. Funding and resources were provided by provincial partners, HH, and CU-PT. These contributions covered essential program needs, including training materials, training certificates, transportation for facilitators and participants, lodging for facilitators and participants, and food. The use of existing community spaces helped reduce costs and ensure that the program remained accessible to participants. Although no direct financial compensation was provided for participants to attend the training, their participation was supported by their employers.

#### The action: strategy development and instructional design

Development of MCM was guided by core principles of implementation science. Rather than introducing a new clinical intervention or protocol, MCM was intentionally designed as an implementation strategy aimed at increasing the integration of evidence-based movement and physical activity practices (assessment, counseling, and prescription) into participants’ workflow. This approach aligns conceptually with the Capability-Opportunity-Motivation (COM-B) model, which posits that behavior change requires sufficient capability, opportunity, and motivation to be successful. (18) In this study, MCM targeted capability through experiential learning; opportunity by embedding the training within existing provincial and health system structures, and motivation by grounding content in locally identified priorities. The initial concept for MCM emerged through collaborative dialogue with local leaders from Pichincha and Cotopaxi Provinces. Through appreciative inquiry (19) and asset mapping (20) we constructed a shared understanding of priorities (e.g., heart disease, stroke, hypertension, type 2 diabetes, arthritis, and back pain), assets (healthcare providers and community health workers), and training needs (e.g., injury/disease prevention and health promotion).

Indigenous community members in Pichincha and Cotopaxi Provinces tend to be very physically active; however, such activity is often aerobic in nature (e.g., walking). Declining strength, flexibility, and balance over time contributes to age-related fall risk and declines in physical activity. “Get Fit for Life”, published by the United States National Institute on Aging, emphasizes the development and maintenance of the four essential components of movement that support physical activity: strength, flexibility, balance, and endurance (i.e., aerobic activity) (21). This evidence-based intervention was selected to provide foundational content. Its focus on functional activities within everyday life further aligned with partners’ vision for MCM. WHO guidelines (12) informed minimum strength and endurance recommendations, while the American College of Sports Medicine guidelines (22) informed the development, progression, and maintenance of these activities.

Adult learning theory (23) and Freirean pedagogy (24) informed all learning activities. Paulo Freire’s approach emphasizes mutual respect, dialogical co-learning, and transformative action. Rather than depositing information into passive learners, Freire advocated for pragmatic, problem-based activities grounded in real-world challenges. Such ongoing and critical reflection strengthens learners’ capacity to apply insights to their daily practice. Table 1 summarizes key learning objectives and activities for the 2 groups.

**Table 1:** Training objectives and learning activities by group.

Group 1 (Action targets: MDs, nurses, physios, and other ministry of health professionals) 1-day × 6 h workshop		Group 2 (Action targets: Community health workers and physios) 2-day × 6 h workshop	
Objectives	Learning activities (Actions)	Objectives	Learning activities (Actions)
Examine the relationship between movement and health	Collaborative conversations: What is movement? What forms of movement are part of everyday life, work, or tradition? What forms of movement bring joy, meaning, or connection? If movement is like medicine, what does it help?	Explore what it means to be healthy	Collaborative conversations and critical reflection: What does it mean to be healthy in your community? What traditional practices support health and well-being? What gets in the way of being healthy? Lived experiences and expertise: Do national mortality and morbidity data resonate with you and what you have seen in your community? What are the priority health issues in your community?
Describe the four components of movement and summarize key principles underlying each	Co-construction: For each component, participants co-created a shared definition, discussed its role in supporting movement and health, and identified contextual examples.	Examine the relationship between movement and health	Collaborative conversations: What is movement? What forms of movement are part of everyday life, work, or tradition? What forms of movement bring joy, meaning, or connection? If movement is like medicine, what does it help?
Consider simple movement prescriptions within the context of your work	Problem-based learning: teams of 3 participants selected 1 of 3 cases and explored what movement components might be important to consider and promote.	Describe the four components of movement and summarize key principles underlying each.	Co-construction: For each component, participants co-created a shared definition, discussed its role in supporting movement and health, and identified contextual examples.
Explore the role of physiotherapists as movement experts	Lived experiences and expertise: What have you seen? What falls within professional scope of practice in Ecuador? What can you imagine?	Practice sample activities within each component	Experiential learning: Participants practiced each activity and were encouraged to perceive how each movement felt in their own body. Lived experiences and expertise: What of these activities feel most appropriate for members of the community?
		Explain the role of posture and body position in performing activities safely Consider simple movement prescriptions within the context of your work.	Practice-based learning: Participants reviewed functional postures and safe lifting techniques for agricultural workers and caregivers. Teams selected 1 of 3 cases and explored what movement components might be important to consider and promote.

#### Instructor qualifications

The instructors (i.e., the implementation actors who delivered the strategies) bring a blend of community connections, linguistic and cultural perspectives, logistical experience, content knowledge, and cultural humility that helped shape both the development and delivery of MCM. Two instructors (PB and CQ) have a long history of collaboration with community leaders and intimate knowledge of traditional customs and practices. The other two instructors (DM and LW) join as external partners from the United States whose experience in transformative community engagement, movement, and health promotion was brought forward to complement local perspectives. Materials and instruction were provided in Spanish, with Quechua interpretation and translation as needed.

### Outcome measures, data collection, and analysis

This early-stage evaluation focused on core implementation outcomes: acceptability, appropriateness, and feasibility (25, 26). Participants indicated their level of agreement, via survey, with several acceptability, appropriateness, and feasibility statements. Given the exploratory nature of this study, a threshold of 80% agreement was established *a priori* to suggest strong endorsement of the study’s implementation outcomes. Proximal adoption of the program was measured by pre-post changes in providers’ confidence. Differences were analyzed using a two-tailed Wilcoxon Signed-Rank Test for paired, non-parametric data. Qualitative data were collected via text responses to open-ended survey questions and analyzed via deductive thematic content analysis (17). Such data collection methods were preferred over interviews or focus groups given the study’s exploratory nature and limited resources. Qualitative responses were examined alongside quantitative results to help contextualize findings. Please refer to Table 2 for a list of measures, definitions, and corresponding quantitative and qualitative data sources.

**Table 2:** Summary of measures and corresponding data sources.

Category	Quantitative measures	Qualitative measure
Participant demographics	Role length of time in current position	Not applicable
Implementation outcomes		
Acceptability *How well the training was received*	Agreement with statements regarding the program’s objectives, facilitators, learning environment, pace, and materials	What additional comments or suggestions do you have? What topics or activities should we include in future workshops?
Appropriateness *Perceived relevance of the training*	Agreement with statements regarding the program’s content, cultural-responsiveness, and relevance	What aspect of the workshop was most useful? What aspect of the workshop was least useful or needs improvement?
Feasibility *The extent to which strategies can be used in a given setting*	How likely are you to integrate these strategies into your practice? What specific strategies will you integrate?	Not applicable
Confidence	Level of confidence pre-post workshop: identifying components of movement; discussing the benefits of movement; exploring community beliefs regarding movement and health; demonstrating simple movement activities; making movement recommendations.	Not applicable

## Results

Findings support the acceptability, appropriateness, and feasibility of a one or two-day movement-based workshop designed for healthcare providers and community health workers. In addition, the program was effective in increasing participants’ confidence regarding movement and movement prescription, a proximal indicator for program adoption.

### Participant demographics

Fifteen participants completed the one-day workshop and 21 participants completed the two-day workshop (Table 3). The majority of participants at the one-day workshop were healthcare providers with more than 5 years of experience. The majority of participants at the two-day workshop were CHWs with more than 5 years of experience.

**Table 3:** Participant demographics.

Characteristic	Group 1 *N* = 15	%	Group 2 *N* = 21	%
Role within the health system				
Community health worker	0	0	18	86
Healthcare provider	13	86	3	14
Administrator	1	7	0	0
Other	1	7	0	0
Years of experience in current role				
<1 year	0	0	0	0
1–3 years	2	14	1	5
3–5 years	1	7	3	14
>5 years	12	79	17	81

### Acceptability, appropriateness, and feasibility

Table 4 provides a joint display of the program’s perceived acceptability, appropriateness, and feasibility. It is worth noting that participants of the two-day workshop tended to rate the program more favorably; however, these differences were not statistically significant (data not shown). The mean acceptability score across training groups was 4.8 (Standard Deviation = 0.69). Ninety percent or more of participants strongly agreed that the training objectives were clearly stated, the facilitators were knowledgeable about the topics and created an engaging learning environment, and the pace of the training was appropriate. Eighty percent of participants strongly agreed that the training materials supported their learning. Many participants remarked on the quality of the training and indicated an interest in future trainings.

**Table 4:** Joint display of acceptability, appropriateness, and feasibility.

Implementation Outcome	Mean	SD	Min	Max	% SA	% A/SA
Acceptability						
The training objectives were clearly stated	4.9	0.3	4	5	90	100
The facilitators were knowledgeable about the topics	4.87	0.72	2	5	97	97
The facilitators created a safe, respectful, and engaging learning environment	4.79	0.76	1	5	90	97
The pace of the training was appropriate	4.83	0.73	1	5	93	97
The visuals, handout, and demonstrations supported my learning	4.7	0.78	1	5	80	97
Qualitative themes - acceptability	Most participants remarked on the quality of the training. *Excelente taller, mejora los conocimientos de las personas que trabajamos en comunidad (Excellent workshop - it improves the knowledge of those of us who work in the community)* There is interest in future trainings, especially for health center staff. *Por favor, siguir capacitando al personal del centro de salud de Saquisili (Please continue providing training to the staff at the Saquisili health center)*					
Appropriateness						
The training content was relevant to my work	4.9	0.4	3	5	93	97
The training included examples that were culturally appropriate for our community	4.67	0.79	1	5	77	97
The program increased my understanding of “Movimiento como Medicina”	4.83	0.75	1	5	93	97
Qualitative themes - appropriateness	What topics or activities should we include in future workshops? Many participants reflected on a desire to adapt activities for specific populations: children, individuals who are pregnant, people with disabilities. There was also interest in designing programs to support individuals with specific health conditions: obesity, stroke, heart disease, osteoarthritis, diabetes, amputation. What aspect of the workshop was most useful? Talking through and applying content to learning cases and practicing various strength, flexibility, and balance activities. *La práctica para conocer los ejercicios que se deben realizar (practice to become familiar with the exercises that need to be performed)*. Clarity of explanations and examples *Explicación dinámica en cada elemento (dynamic explanation of each element)* What aspect of the workshop was least useful or needs improvement? Most participants did not identify areas for improvement; however, the following perspectives were shared: Spend less time reviewing theory and more time practicing (*abarcar la parte de conceptos*) Pay more attention to ergonomics (*conceptos de ergonómica*) and balance activities Incorporate examples of patients who are bedridden or who have disabilities (*Utilización de ejemplos en pacientes encamados*)					

Feasibility	Mean	SD	Min	Max	% VL	% L/VL
How likely are you to incorporate movement-based strategies into your practice after this training?	4.66	0.61	3	5	75	97
Which of the following behaviors will you incorporate into your practice?	%					
Asking clients about their movement and physical activity	59					
Teaching patients simple movements they can do at home	66					
Recommending movement to prevent or manage chronic conditions	83					
Connecting patients with community-based resources	21					
Using MCM during educational sessions	72					

SD, standard deviation; Min, minimum; Max, maximum; SA, strongly agree; A, agree; VL, very likely; L, likely.

The mean appropriateness score was = 4.8 (Standard Deviation = 0.68). Ninety-three percent of participants strongly agreed that the content was relevant to their work and that the program increased their understanding of movement as medicine. Seventy-seven percent of participants strongly agreed that the training included culturally appropriate examples. The most useful or relevant aspects of the training included talking through and applying content to learning cases and practicing the various movement-based strategies. The least useful or relevant aspect of the training was the review of concepts and theory. Participants recommended that the training devote more time to reviewing ergonomics, practicing balance strategies, and adopting strategies for special populations (e.g., children, individuals who are pregnant, people with disabilities, individuals with specific health conditions).

The mean feasibility score was 4.65 (Standard Deviation = 0.61). 97% of participants were likely or very likely to integrate movement strategies into their practice as a result of the workshop. Frequently endorsed strategies included recommending movement to prevent or manage chronic conditions (83%), teaching simple home-based movement activities (66%), using the “Movimiento como Medicina” elements in community-based educational sessions (72%), and asking patients and community members about their movement (59%).

### Proximal adoption

Participants’ confidence in identifying the four components of movement increased significantly from pre- to post-training across groups (Table 5). Confidence among Group 2 participants also significantly increased in terms of their ability to discuss the benefits of movement, explore community beliefs regarding movement, demonstrate simple movement-based activities, and make safe and practical movement recommendations. It is worth noting that pre-training confidence among participants of the two-day workshop was significantly lower than pre-training confidence among participants of the one-day workshop, with the exception of their confidence in identifying the four components of movement. Post-training confidence among participants was similar across groups.

**Table 5:** Proximal adoption: change in participant confidence pre-post training.

Behavior	Mean pre (SD)	Mean post (SD)	Mean difference (95% CI)	*p*-value (two-tailed test)
Identifying the 4 components of movement				
Group 1	3.4 (0.7)	4.5 (0.4)	1.1 (0.4–1.7)	0.004*
Group 2	3.3 (1.2)	4.8 (0.4)	1.5 (0.9–2.1)	<0.001*
Discussing the benefits of movement for chronic disease prevention and management				
Group 1	4.4 (0.8)	4.6 (0.6)	0.2 (−0.3–0.6)	0.5
Group 2	3.3 (1.0)	4.8 (0.4)	1.5 (1.0–1.9)	<0.001*
Exploring community beliefs regarding movement and health				
Group 1	4.5 (0.8)	4.6 (0.6)	0.1 (−0.3–0.7)	0.336
Group 2	3.3 (0.9)	4.6 (0.5)	1.3 (0.8–1.7)	<0.001*
Demonstrating simple movement-based activities				
Group 1	4.6 (0.7)	4.6 (0.6)	0.0 (−0.2–0.5)	0.435
Group 2	3.3 (0.9)	4.4 (0.5)	1.1 (0.8–1.6)	<0.001*
Making safe and practical movement recommendations				
Group 1	4.5 (0.7)	4.8 (0.4)	0.3 (−0.07–0.8)	0.096
Group 2	3.5 (0.8)	4.4 (0.6)	0.9 (0.5–1.7)	<0.001*

SD, standard deviation; 95% CI, 95% confidence interval.

*Significant at *p* < 0.05 (two-tailed test).

## Discussion

Despite Ecuador’s emphasis on health and physical activity promotion, there is limited evidence regarding the design, implementation, and evaluation of community-based training programs that can be embedded within Indigenous-serving health systems. Findings from this pilot project demonstrate that *Movimiento como Medicina* (MCM) is a highly acceptable, appropriate, and feasible implementation strategy for strengthening the capacity of health professionals in the Ecuadorian highlands to promote evidence-based movement and physical activity recommendations. Significant gains in providers’ confidence, an important precursor to consistent behavior change, serve as a proximal indicator of program adoption. This is further supported by the high proportion of participants who reported being likely or very likely to implement movement-based strategies within their daily practice.

### Physical activity promotion in Ecuador: an implementation gap

This study fills an important implementation gap within Ecuador’s Indigenous health promotion landscape. Studies exploring the promotion of movement and physical activity in Ecuador are primarily focused on intervention effectiveness in school-based settings (27, 28). For example, a preschool-based program was implemented in nine municipal preschools in Cuenca, a large city in the Andean highlands with approximately 500,000 people. Children enrolled in the program demonstrated improvements in their dietary behaviors, screen time, and body mass index over the 10-month study period (27). Similarly, the ACTIVITAL program, also implemented in Cuenca, employed educational and environmental strategies within secondary schools to increase physical activity and decrease screen time among adolescents (28). The proportion of students achieving over 60 min of moderate-to-vigorous physical activity per day decreased the study period, though this decline was smaller in intervention schools compared to control schools (6% versus 18%). No significant differences were noted in screen time or BMI.

While these studies provide useful evidence regarding intervention outcomes within school-based settings, they offer limited insight regarding core implementation outcomes and workforce training preferences within existing provincial and healthcare systems. Evidence of movement-based training programs developed for healthcare providers in Ecuador is sparse. One exception is Exercise is Medicine, a one-day workshop developed by the American College of Sports Medicine and the American Medical Association (29). This one-day workshop covered the health benefits and risks associated with physical activity, patient screening and risk stratification, and general principles of exercise prescription. Reported gains among Ecuadorian providers were minimal, perhaps highlighting potential implementation challenges related to contextual fit, cultural appropriateness, and feasibility.

We are not aware of any studies describing movement-based programs led by CHWs in Ecuador; however, evidence from Indigenous communities in Australia and Canada suggests that CHWs are well-positioned to deliver this content (30). In Queensland Australia, the B.Strong program sought to build CHW capacity through the provision of a one-day workshop, six online modules, and a resource toolkit. Twelve culturally-appropriate, plain language brochures were co-developed with CHWs across three domains: Quit for Health; Eat for Health; and Move for Health. Participants demonstrated significant gains in knowledge, confidence, and the delivery of brief interventions across these domains. Similarly, Makoyoh’sokoi (The Wolf Trail Program), an 18-week wellness program created by and for Indigenous women in the Alberta and Saskatchewan provinces of Canada (31), illustrates the power of a community-led approach that honors Indigenous epistemologies and relational learning. Taken together, these studies underscore the importance of implementation strategies that prioritize workforce development, community ownership, and cultural alignment.

### Culturally responsive implementation

Across these five programs, a continuum has emerged in terms of the extent to which culturally-responsive and participatory implementation strategies were used. At one end of the spectrum, the school-based (27, 28) and physician-focused interventions (29) prioritized knowledge transmission (i.e., “banking”), with limited evidence of critical dialogue, co-design, or participatory evaluation (27, 28). These programs demonstrated effectiveness in improving short-term knowledge and individual-level behavior change, but largely reflect a researcher-driven model, rooted in Western biomedical paradigms.

Workforce strategies, such as the B.Strong program (30), occupy the middle of this continuum. On the one hand, it centered the program around Indigenous health workers, emphasizing the importance of relational communication and acknowledging contextual constraints. On the other hand, the program was primarily competency-based and instructional, with limited opportunities for problem-based learning, knowledge construction, or participatory evaluation. Cultural responsiveness was present in terms of who the program was designed for and where it was delivered, but less evident in how learning occurred.

On the other end of the spectrum is the Makoyoh’sokoi (Wolf Trail) program (31) which exemplifies deep cultural-responsiveness and Freirean alignment. This community-led, movement-based wellness program was grounded in Indigenous epistemologies from inception, employed dialogical and relational learning processes, and framed movement as a collective, land-based and identity-affirming practice. Evaluation methods centered Indigenous definitions of wellness and emphasized the power of personal narratives, collective story-telling, and connection.

MCM likely falls in the middle of this continuum. The following contextually grounded implementation methods likely contributed to its high ratings:

- Completing an asset-based community health assessment grounded in appreciative inquiry
- Building a coalition with partners from HH, Gobierno Provincial de Cotopaxi and Cotopaxi Solidario, and Ministerio de Salud Publica de Pichincha
- Embedding the program within existing organizations and structures
- Co-creating learning objectives
- Leveraging Freirean pedagogy to inform all learning activities

While acceptability, appropriateness, feasibility, and proximal adoption scores were high, only 80% of participants strongly agreed that the materials supported their learning and 77% of participants strongly agreed that the training included culturally appropriate examples. Despite leveraging numerous contextually grounded strategies, the topics and evaluation framework ultimately reflected the biomedical and public health traditions of the authors. In looking ahead to the next phase of *MCM*, participant appraisals support the elevation of community-led approaches, including (1) better grounding within Indigenous knowledge systems and traditional movement practices, (2) personal narratives and collective story-telling, and (3) evaluation frameworks that prioritize community-defined outcomes.

### Future directions

The high acceptability, appropriateness, feasibility, and proximal adoption of MCM suggest it has the potential to be scaled as part of a regional or national CHW and health provider implementation strategy. Future research should prioritize a deeper exploration and integration of Indigenous knowledge systems and traditional movement practices to ensure cultural relevance while strengthening community ownership. Planned next steps include equitable engagement with local Indigenous leaders, healers, and CHWs in co-designing workshops to collaboratively define what it means to be healthy, identify culturally-meaningful movement practices, create relevant learning cases, and consider community-driven outcomes. Expanding the program to include other health behaviors (e.g., nutrition) within their social and structural context, may provide a more holistic approach to health promotion. Finally, future efforts should examine the effectiveness of the program in changing provider behaviors. Gamification and mobile health (mHealth) tools may prove especially useful in measuring engagement and behavior change. Assessing resource requirements will be critical in informing future policy and funding decisions. Enhancing community ownership through participatory co-design can position MCM to promote movement, not merely as an individual-level behavior, but as a culturally embedded, collective practice that supports community agency, connection, and long-term health.

### Limitations

This exploratory study has several limitations that should be considered when interpreting its findings. First, recruitment methods may have introduced selection bias by attracting individuals already interested in health promotion and movement-based interventions. Related, the sample was drawn from a relatively small geographic region, which may not reflect the cultural knowledge, beliefs, or practices of other Indigenous communities. Such selection and geographical biases likely limit the study’s generalizability. Second, although efforts were made to ensure cultural and linguistic appropriateness by collaborating with bicultural and bilingual partners, the program was primarily informed by Western frameworks and guidelines (e.g., WHO and ACSM). Such frameworks do not reflect Indigenous knowledge systems, nor do they address the many structural drivers of health that are rooted in colonial legacies. Third, the intervention was brief - one or 2 days depending on the group - and lacked long-term follow-up, making it difficult to determine whether increased confidence translated into sustained practice changes or improved community health outcomes. The program’s focus on movement, while intentional, excluded other critical health behaviors, which may limit the program’s overall impact on chronic disease prevention and management. Fourth, outcome measures relied on self-reported data, which are subject to social desirability bias. Moreover, the use of an 80% agreement threshold for acceptability and applicability is somewhat arbitrary and may not capture nuanced participant feedback. We sought to address this issue through the use of open-ended questions and text responses.

## Conclusion

This pilot study of *Movimiento como Medicina* (MCM) addresses a critical gap in Ecuador’s Indigenous health promotion landscape and centers CHWs and local healthcare providers as important catalysts for change. The program’s high acceptability, appropriateness, and feasibility, combined with significant improvements in participant confidence, positions MCM as a contextually aligned and scalable health promotion strategy. Comparisons with Indigenous-led programs in Australia and Canada highlight opportunities for MCM to evolve through a stronger, more intentional approach to co-design that elevates Indigenous knowledge systems and traditional movement practices.

## Data Availability

The raw data supporting the conclusions of this article will be made available by the authors, without undue reservation.
